# Second eyes to develop neovascular age-related macular degeneration have fewer symptoms and better one-year visual outcomes

**DOI:** 10.1186/s12886-023-03021-0

**Published:** 2023-07-07

**Authors:** F. Sema Akkan Aydoğmuş, Oluchukwu Onwuka, Jackson Saddemi, Claudia C. Lasalle, David J. Ramsey

**Affiliations:** 1 Ophthalmology Department, Ankara Sehir Hastanesi, Üniversiteler, Çankaya, Ankara, Turkey; 2 Department of Ophthalmology, Lahey Hospital & Medical Center, 1 Essex Center Drive, Peabody, MA 01960 USA; 3 Department of Ophthalmology, Tufts University School of Medicine, Boston, MA 02111 USA; 4 Cooper Medical School, Rowan University, Camden, NJ 08103 USA

**Keywords:** Neovascular age-related macular degeneration, Anti-vascular endothelial growth factor, Choroidal neovascularization, Fellow eye, Risk factors, Retinal pigment epithelium detachment, Optical coherence tomography, Visual acuity

## Abstract

**Background:**

This study compares the visual and anatomical outcomes for the eyes of patients who developed sequential neovascular age-related macular degeneration (nAMD), both at the time of diagnosis and at one year after treatment.

**Methods:**

The study comprised a retrospective case series of 52 patients whose eyes were diagnosed sequentially with nAMD. All eyes were treated with three monthly loading doses of anti-vascular endothelial growth factor agents, followed by further intravitreal injections, as required. Baseline characteristics and outcomes at one year after diagnosis and initial treatment were compared between first and second eyes and included visual acuity (VA), central macular thickness (CMT), and pigment epithelial detachment (PED) height on optical coherence tomography (OCT) imaging.

**Results:**

VA at diagnosis was better for second eyes compared with first eyes to develop nAMD (logMAR 0.68 ± 0.51 versus logMAR 0.41 ± 0.34, *P* = 0.002) and remained so at one year (logMAR 0.61 ± 0.60 versus logMAR 0.42 ± 0.37, *P* = 0.041). Similarly, PED height at diagnosis was higher in first eyes (225 ± 176 μm versus 155 ± 144 μm, *P* = 0.003) and also at one year (188 ± 137 μm versus 140 ± 112 μm, *P* = 0.019). Whereas most patients reported symptoms at first eye diagnosis (71.2%), half as many second eyes were symptomatic (28.8%, *P* < 0.001). Significantly more symptomatic first eyes experienced visual distortions (32.4% versus 13.3%) or scotomas (29.4% versus 6.7%), compared with a less specific visual complaint of blurry vision (38.2% versus 80.0%, *P* = 0.006).

**Conclusions:**

Compared with first eyes to develop nAMD, second eyes tended to have better vision, smaller PED heights, and fewer symptoms likely because monitoring permitted earlier diagnosis.

## Introduction

Neovascular age-related macular degeneration (nAMD) is the most common, progressive disease that results in severe vision loss among individuals 50 years of age and older in high-income countries [1]. nAMD most often starts in one eye, with 18–24% of at-risk, fellow eyes going on to develop the condition within two years [2–8]. The frequent visits required to evaluate and manage nAMD in one eye provide an opportunity to surveil fellow eyes for signs of active choroidal neovascularization (CNV), which is the hallmark of nAMD. Monitoring the fellow eye at these visits by means of optical coherence tomography (OCT) imaging often results in the diagnosis of nAMD at less advanced stages of disease when eyes have better visual acuity (VA) [2, 3] and fewer symptoms attributable to the disease [8–10]. Thus, early detection of CNV before lesions progress to cause vision loss may make it possible to maintain vision-related quality of life [11–13].

In the present study, we compared the visual and anatomical outcomes for the eyes of patients who developed sequential nAMD, both at the time of diagnosis and after one year of treatment. Specifically, we assessed characteristics associated with nAMD severity, including the type and extent of reported symptoms, location and degree of retinal fluid, clinical evidence of blood, and presence and height of any associated pigment epithelial detachment (PED) detected by means of OCT imaging. We sought to determine if any of these features correlated with the number and frequency of intravitreal injections (IVIs) received, or likelihood that eyes would be free of disease activity after one year of treatment.

## Methods

This research followed the tenets of the Declaration of Helsinki. The Institutional Review Board of the Lahey Hospital & Medical Center approved the study and provided a waiver of the requirement for informed consent because of the retrospective nature of the study. All data was gathered and secured in compliance with the Health Insurance Portability and Accountability Act.

### Electronic health record data extraction

The study comprised a retrospective case series of patients whose eyes were diagnosed sequentially with nAMD between March 2015 and December 2020. All eyes were treatment-naïve when diagnosed. A gap of more than 31 days from the clinical diagnosis of nAMD in the first eye to the second eye was used as the definition of sequential nAMD [2]. All eyes were treated with an initial series of three-monthly loading doses of anti-vascular endothelial growth factor agents with subsequent management intervals determined by the physician following a treat-and-extend protocol, with the goal of maintaining a fluid-free macula [10, 14]. All eyes included in the study had one year or more of uninterrupted follow-up after the diagnosis of nAMD. Patients whose eyes stopped treatment after three IVIs because of documented treatment failure, who underwent ancillary treatments (e.g., photodynamic therapy), or had poor central vision (VA < 20/300) in the fellow eye were excluded. Additionally, individuals with other retinal conditions (e.g., retinal vein occlusions, diabetic retinopathy, myopic degeneration, or central serous retinopathy) were excluded. Patients who had cataract surgery within three months before diagnosis of nAMD, had a history of any prior retinal surgery, or received treatment at an outside facility were also excluded.

A retina specialist reviewed the clinical and demographic data, including family history of AMD, frequency of IVIs, and the use of Age-Related Eye Disease Study (AREDS) vitamins, or their equivalent. Severity of AMD was based on the International Classification of Diseases, Tenth Revision, Clinical Modification (ICD-10-CM) codes. Symptoms reported on the date of nAMD diagnosis, if any, were also recorded and assigned to one of three categories: decreased vision, visual distortion/metamorphopsia, or report of a scotoma. Data derived from spectral domain OCT images of the macula (Cirrus [Carl Zeiss Meditec, Inc] or Spectralis [Heidelberg Engineering, Inc, Heidelberg, Germany]) included automated central macular thickness (CMT), as well as manual notation of any subretinal fluid (SRF), intraretinal fluid (IRF), and maximum height of retinal PED, if any. For the purposes of analysis, PEDs were defined as a separation of at least 50 μm between the retinal pigment epithelium and inner aspect of Bruch’s membrane [15].

### Statistical analysis

Data were analyzed using SPSS (version 28.0, IBM, Armonk, NY, USA). Snellen VA was converted to the logarithm of the minimum angle of resolution (logMAR), and CMT heights from scans obtained on the Cirrus platform were adjusted to allow for comparison with scans obtained on the Heidelberg platform for statistical analysis [16]. The Chi-square test or Fisher’s exact test were used to assess the difference between categorical variables. Continuous variables were recorded as mean ± standard deviation (SD) and were analyzed using the Student’s *t*-test for normally distributed variables and Mann–Whitney U test for non-normally distributed variables based upon the Shapiro–Wilk test. Pearson’s correlation coefficient was used to assess for any association between continuous variables. All tests were two-sided, and *p*-values below 0.05 were considered statistically significant.

## Results

Fifty-two patients who sequentially developed nAMD met inclusion criteria. The mean age of patients at diagnosis of the first eye was 81.6 ± 8.0 years; 65% of patients were female and 48% were initially diagnosed in their right eyes (Table 1). The average time between diagnosis of the first and second eye with nAMD was 440 ± 328 days (16 months) with first eyes receiving an average of 8.1 ± 6.3 IVIs (range 1 to 33 IVIs) before second eye conversion. Fluorescein angiography (FA) was performed at diagnosis for 23% of first eyes and 7.7% of second eyes (χ^2^ = 4.7273, *P* = 0.030). The time from last retinal evaluation to second-eye diagnosis was 68 days ± 64 days. Prior to being diagnosed with nAMD, most fellow eyes were identified to have intermediate-stage AMD (69%), with far fewer eyes having early (23%) or advanced dry AMD (7.7%). Notably, 73% of patients reported taking AREDS vitamins or an equivalent supplement.

**Table 1:** Baseline demographic and clinical characteristics for patients who developed sequential neovascular age-related macular degeneration

Characteristic	
**Age at diagnosis, years**	
First eye, mean (SD)	81.6 (8.0)
Median (range)	83 (60–94)
Second eye, mean (SD)	82.8 (8.1)
Median (range)	84 (60–96)
**Sex, n (%)**	
Female	34 (65.4)
**Race, n (%)**	
White (non-Hispanic)	50 (96.1)
**First treated eye, n (%)**	
Right eye	25 (48.1)
**Time between diagnosis of the** **first and second eye, days**	
Mean (SD)	440 (328)
Median (range)	362 (32–1356)
**Disease severity in fellow eye** **at first eye diagnosis, n (%)**	
Early	12 (23.1)
Intermediate	36 (69.2)
Advanced	4 (7.7)
**Sociomedical characteristic, n (%)**	
Nutritional supplement use^1^	38 (73.1)^2^
Active smoker	2 (3.8)
Former smoker	16 (30.8)
No smoking history	34 (65.4)
Family history of AMD	18 (34.6)

^1^Includes use of Age-Related Eye Disease Study (AREDS) and/or AREDS 2 vitamins, or their equivalent. ^2^Two additional Patients started AREDS 2 vitamins by the time of second eye conversion

### Treatment characteristics

The mean number of IVIs delivered in the first year of treatment was similar for the first compared with second eyes (8.7 ± 2.4 injections versus 9.0 ± 2.0 injections, *P* = 0.699). Seven first eyes to develop nAMD were switched from bevacizumab to aflibercept (13.5%). A similar number of second eyes to develop nAMD were either switched from bevacizumab to aflibercept (9.6%) or started on aflibercept at diagnosis (5.8%), for a total eight second eyes (15.4%, χ^2^ = 0.2955, *P* = 0.587). Of note, the time to switch in agent was similar for the first compared with second eyes, excluding those eyes that were started on aflibercept (249 ± 85 days [range 105 to 343 days] versus 210 ± 78 days [range 126 to 337 days], *P* = 0.425). At one year, the average injection interval was 55.6 ± 27.8 days for first eyes compared with 57.1 ± 26.1 days for second eyes (*P* = 0.764); a small number of eyes (13.5% of both groups) remained on treatment at monthly intervals one year after diagnosis. There was no correlation between IVI interval, total number of IVIs, or type of agent and disease activity at one year. No participant included in the study had any serious systemic or ocular adverse events reported.

### Baseline characteristics

Only slightly more than half of first eyes to develop nAMD had a documented VA available within the year prior to the diagnosis of nAMD (61.5%). For this subset of patients, there was no difference in baseline vision prior to the diagnosis of nAMD in the first compared with the second eye to develop the disease (logMAR 0.28 ± 0.28 versus logMAR 0.24 ± 0.23, *P* = 0.621). Eyes lost vision after being diagnosed with nAMD (average visual acuity decreased to logMAR 0.64 ± 0.46 [*P* < 0.001] for first eyes and to logMAR 0.41 ± 0.34 [*P* < 0.001] for second eyes, respectively). However, there was a smaller decline in vision for the second eyes to develop the disease (∆logMAR 0.36 ± 0.41 versus ∆logMAR 0.17 ± 0.29, *P* < 0.001). In total, 42 first eyes (81.3%) and 34 second eyes (65.4%) had a reduction of one or more lines in VA after the development of nAMD (χ^2^ = 0.0911, *P* = 0.763).

Imaging data were available for 23 first eyes (44.2%) and for all 52 second eyes prior to the diagnosis of nAMD. Prior to diagnosis of nAMD the average CMT of these first eyes was similar to that of second eyes (264 ± 32 μm versus 259 ± 40 μm, *P* = 0.652). The increase in CMT after the diagnosis of nAMD was also similar for these first eyes compared with second eyes (∆CMT 61 ± 59 μm versus ∆CMT 59 ± 69 μm, *P* = 0.742).

### Comparison of first with second eyes to develop nAMD

VA at diagnosis was significantly better for the second eyes compared with first eyes to develop the disease (logMAR 0.68 ± 0.51 versus logMAR 0.41 ± 0.34, *P* = 0.002; Table 2). Despite similar treatment, the visual outcomes were significantly better for second eyes compared with the first eyes after one year of treatment (0.61 ± 0.60 logMAR versus 0.42 ± 0.37 logMAR, *P* = 0.041). The change in vision for first eyes weakly correlated with the number of IVIs received (r=-0.383, *P* = 0.005). This was not the case for second eyes to develop the disease (r=-0.093, *P* = 0.512), likely in part because these eyes started with better vision, leaving less room for visual gains. In summary, after one year of treatment, 42 first eyes (80.8%) either maintained or gained vision, with 22 (42.3%) gaining three or more lines of vision. By comparison, 34 second eyes (65.4%) either maintained or gained vision (χ^2^ = 3.128, *P* = 0.077), with significantly fewer (six eyes, 11.5%) gaining three or more lines of vision (χ^2^ = 12.511, *P* < 0.001). Only five first eyes (9.6%) and six second eyes (11.5%) lost three or more lines of vision (χ^2^ = 0.1017, *P* = 0.750).

**Table 2:** Clinical characteristics and anatomical outcomes at diagnosis and at one year for eyes diagnosed with sequential neovascular age-related macular degeneration

Characteristic	Groups		
	**First Eye**	**Second Eye**	***P-value*** ^1^
**VA, logMAR (SD)**			
At diagnosis	0.68 (0.51)	0.41 (0.34)	***0.002*** ^2^
At 12 months	0.61 (0.60)	0.42 (0.37)	***0.041*** ^2^
***P-value***^1^	*0.241* ^2^	*0.872* ^2^	
**CMT, µm (SD)**			
At diagnosis	340 (92)	311 (73)	0.079^2^
At 12 months	272 (57)	265 (53)	0.467^2^
***P-value***^1^	*<* ***0.001***^2^	*<* ***0.001***^2^	
**Hemorrhage, n (%)**			
At diagnosis	10 (19.2)	6 (11.5)	0.416^3^
At 12 months	1 (1.9)	3 (5.8)	0.618^3^
***P-value***^1^	***0.008*** ^3^	*0.488* ^3^	
**IRF, n (%)**			
At diagnosis	31 (59.6)	37 (71.2)	0.216^4^
At 12 months	26 (50.0)	27 (51.9)	0.845^4^
***P-value***^1^	*0.325* ^4^	***0.044*** ^4^	
**SRF, n (%)**			
At diagnosis	42 (80.8)	16 (30.8)	***<0.001*** ^4^
At 12 months	17 (32.7)	17 (32.7)	*1.00* ^4^
***P-value***^1^	*<* ***0.001***^4^	*0.833* ^4^	
**PED, n (%)**			
At diagnosis	47 (90.4)	46 (88.5)	*0.750* ^4^
At 12 months	44 (84.6)	*44 (84.6)*	1.00^4^
***P-value***^1^	*0.374* ^4^	*0.566*^4^	
**PED, µm (SD)**			
At diagnosis	225 (176)	155 (144)	***0.003*** ^5^
At 12 months	188 (137)	*140 (112)*	***0.019***^4^
***P-value***^1^	*0.322* ^5^	*0.928*^5^	

^1^Significance is marked in bold (*P* < 0.05). ^2^Student’s t-test. ^3^Fisher exact test. ^4^Chi–square test. ^5^Mann–Whitney U test

**Fig. 1:**
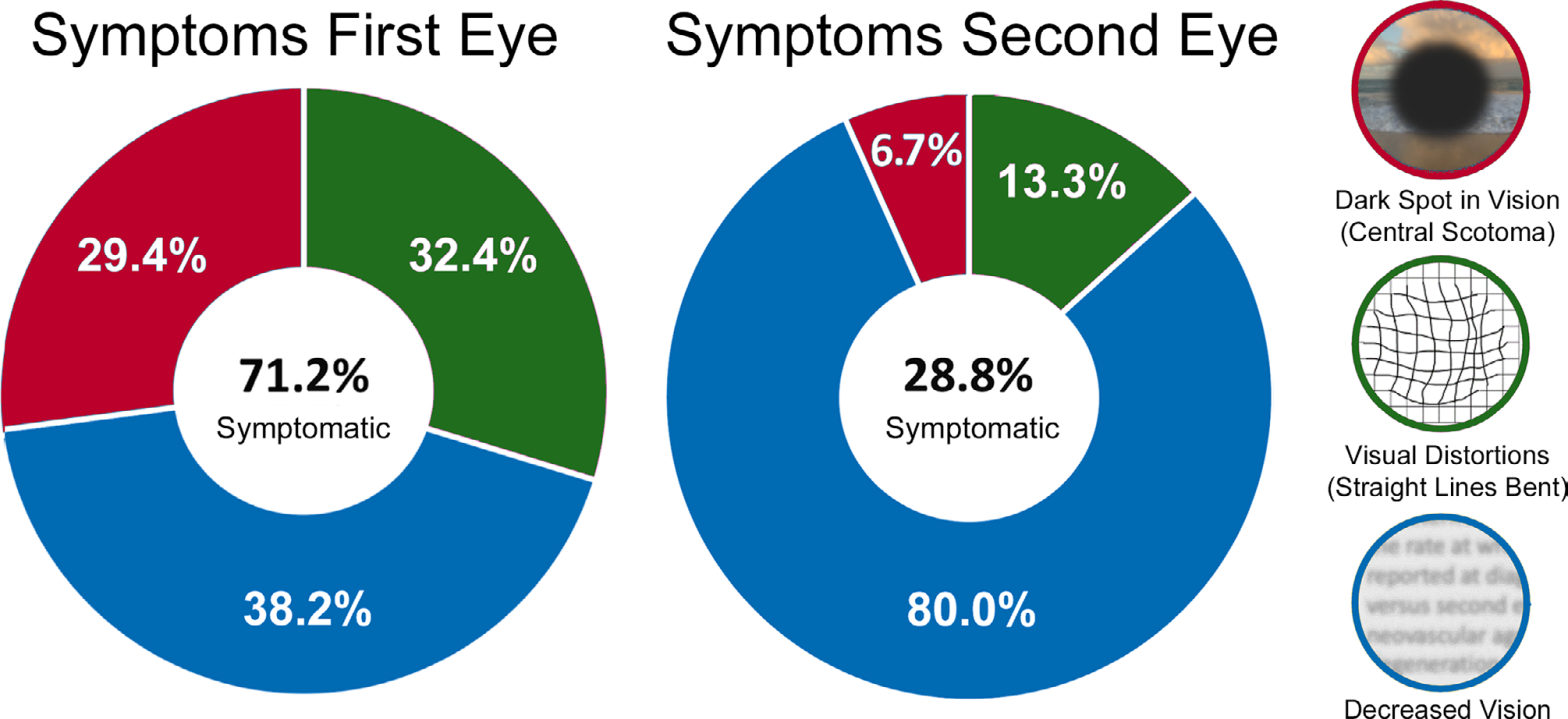
Symptoms reported at diagnosis in patients who developed sequential neovascular age-related macular degeneration. Shown in the center of each graph is the fraction patients who reported symptoms at diagnosis of their first and second eyes, respectively. The anulus displays the type of symptom reported as a percentage of all symptomatic eyes by colour

Whereas most patients reported symptoms likely attributable to nAMD at the time of first eye diagnosis (37 eyes, 71.2%), fewer than *half* as many patients reported symptoms at second eye diagnosis (15 eyes, 28.8%; χ^2^ = 18.615, *P* < 0.001, Fig. 1). Eyes that were asymptomatic were also less prone to vision loss, with fewer than half of all such eyes (41.8%) experiencing a loss of less than one line of vision compared to the previous visit. By contrast, most eyes from patients who reported visual symptoms at the time of nAMD diagnosis demonstrated a reduction of more than one line of vision (79.6%; χ^2^ = 7.675, *P* = 0.006). Furthermore, for this subset of patients who reported symptoms at nAMD diagnosis, significantly more first eyes experienced visual distortions (32.4% versus 13.3%) or scotomas (29.7% versus 6.7%) compared with a less specific visual complaint such as decreased or blurry vision (37.8% versus 80.0%, χ^2^ = 7.589,  P = 0.006). Finally, no specific clinical or demographic feature other than VA correlated with which patients reported symptoms (or the type of symptom reported).

The average CMT at diagnosis (340 ± 92 μm versus 311 ± 73 μm, *P* = 0.079) and at one year (272 ± 57 μm versus 265 ± 53 μm, *P* = 0.467) were similar for the first eyes compared with second eyes to develop nAMD. There was also a similar reduction in CMT at one year in a comparison of all first eyes with second eyes (∆CMT − 62 ± 80 μm versus ∆CMT − 46 ± 80 μm, *P* = 0.171). However, several other clinical and imaging findings varied between the first and second eyes to develop the disease.

Compared with second eyes, first eyes more commonly had SRF at diagnosis (80.8% versus 30.8%, χ^2^ = 26.351, *P* < 0.001). This difference did not remain at one year, by which time an equal number of first and second eyes had SRF detectable on OCT imaging (both 32.7%). By contrast, a similar number of first and second eyes had IRF at diagnosis (59.6% versus 71.2%, χ^2^ = 1.530, *P* = 0.216). Notably, the presence of IRF at one year correlated with worse vision across all study eyes (r = 0.313 *P* = 0.001). Finally, a similar but small number of first and second eyes had a hemorrhage clinically identified in the macula at the time of diagnosis (19% versus 12%, χ^2^ = 1.1818, *P* = 0.277) and at one year (1.9% versus 5.8%, χ^2^ = 1.040, *P* = 0.618). The presence of IRF in the first eye at diagnosis correlated with IRF at diagnosis of the fellow eye (r = 0.514, *P* < 0.001). There was no similar correlation for SRF or macular hemorrhage, two other important structural markers of disease activity. Finally, PED height at diagnosis and at one year were larger for the first eyes compared with the second eyes to develop the disease (225 ± 176 μm versus 155 ± 144 μm [*P* = 0.003] and 188 ± 137 μm versus 140 ± 112 [*P* = 0.019], respectively). Of note, 94.2% of eyes studied had a PED. A modest correlation was identified between PED height at one year and presence of SRF at one year (r = 0.255, *P* = 0.009), as well as worse VA at one year (r = 0.278, *P* = 0.005) for all study eyes.

## Discussion

Among patients who develop sequential nAMD, the second eye diagnosed tends to start with and maintain better VA after one year of treatment compared with the first eye to develop the disease [2–4, 17]. The frequent visits required to evaluate and manage nAMD in one eye provide an opportunity to surveil the fellow eye for signs of the disease. The high sensitivity and specificity of OCT imaging to detect signs of active CNV [9, 18–20] may account for why second eyes in our study tended to be diagnosed while vision was better and PED height was smaller and, in many cases, *before* the disease became symptomatic.

Early diagnosis of nAMD in the second eye is of great importance because these eyes are commonly the patient’s better-seeing eye [2, 3, 17]. Better VA at the time treatment commences is one of the strongest predictors of better long-term visual outcomes [9, 13, 21−25]. In the Comparison of Age-related Macular Degeneration Treatments Trials (CATT) [26], 28% of eyes treated with bevacizumab as needed achieved a ≥ 3-line gain in VA compared with 27% of eyes in our study. However, a significant difference was observed between first- and second-treated eyes in our study, with substantially fewer second eyes achieving ≥ 3-line VA gains. This is likely accounted for by the fact that second eyes had better VA at diagnosis, thereby leaving less opportunity for visual gains at one year because of a ceiling effect [27]. Of note, a similar proportion of eyes in our study compared with CATT maintained VA, with only ~10% of eyes across both studies losing ≥ 3 lines of vision [26].

As in previous studies [27, 28], we found no direct correlation between vision and CMT. However, we found a modest correlation between PED height at one year and worse VA by one year, as well as an association with the presence of IRF at one year. Similar to our study, PEDs in eyes in the HARBOR study decreased in size with treatment; however, PED height at one year was not linked to visual outcomes, as it was for the eyes in our study [29]. Interestingly, a recent study found that treatment-refractory SRF does not lead to long-term vision loss [30]. By contrast, IRF is more closely associated with sustained visual acuity loss [31–34], as was the case in our study.

Few studies have compared the extent to which patients with nAMD report symptoms at diagnosis [8, 17]. The Early Detection of Neovascular Age-Related Macular Degeneration Study [9] reported that 69.2% of patients who developed sequential nAMD failed to note a decrease in vision at second eye diagnosis, which is similar to the rate in our study (71.2%). Not only did we find that symptoms were less likely to be reported, but that the character of those symptoms were less specific at the time of second eye diagnosis. This finding may be related to the fact that in many of the eyes in our study, signs of CNV were detected by routine surveillance with OCT imaging allowing for the diagnosis to be made before eyes had lost substantial vision [9, 10, 18, 35, 36].

Limitations of our study include the relatively small number of patients that met all inclusion criteria and the inclusion of patients whose fellow eyes had different stages of dry AMD. The reliance on Snellen VA, as is the norm in most clinical practices, probably underestimates VA in our study compared with ETDRS vision commonly used in clinical trials [26]. Being a retrospective study, it is also possible that not all symptoms reported by patients were documented, if they were asked at all. Our study population is also derived from a single center, outpatient clinic that serves as a retina referral center. This can account for why some patients lacked historic data for their first eye before being diagnosed with nAMD by a retina specialist. The inclusion of only those patients who had *uninterrupted* follow-up may have selected against patients who had particularly positive or negative outcomes and may not, therefore, be representative of all patients who develop sequential nAMD. Lastly, as is increasingly common in clinical practice [17, 35, 36], not all first eyes and an even smaller number of second eyes in our study underwent FA to define lesion characteristics at diagnosis. Although FA is considered an index test in clinical trials [9, 21–25] the use of this invasive test has decreased over time, especially when confirming involvement of the second eye [37]. However, OCT has a high sensitivity and specificity to detect nAMD and approaches the sensitivity of FA [9, 18]. In the future, optical coherence tomography angiography is likely to become integral to the standard of care because of its ability to identify eyes with CNV lesions even before they produce exudation [19, 38]. Lager studies should also be performed to determine the impact of CNV subtype on the course of sequential nAMD.

## Conclusions

Our study of eyes from treatment-naïve patients who developed sequential nAMD is among the first to examine in detail both the features of nAMD observable by OCT imaging and the impact of those features on visual outcomes at diagnosis and after one year of treatment. Although many retinal characteristics were similar at diagnosis and after one year of treatment, we found that the second eyes to develop nAMD tended to have better vision and smaller PED heights. Understanding the implications of these anatomical features and other disease markers on vision not only benefits clinical practice but sets the stage for future investigations into whether modifying those factors might improve patient outcomes. Finally, at diagnosis, second eyes have fewer and less specific symptoms compared with first eyes. This difference underscores the importance of regular monitoring of fellow eyes of patients with unilateral nAMD to permit earlier diagnosis and treatment at less advanced stages of disease.

## Data Availability

Due to the nature of this research, participants of this study did not agree for their data to be shared publicly, however the data used or analyzed in this study is available from corresponding author on reasonable request.

## Abbreviations

AREDS: Age-Related Eye Disease Study
CATT: Comparison of Age-related Macular Degeneration Treatments Trials
CMT: Central macular thickness
CMT: Central macular thickness
ICD-10-CM: International Classification of Diseases, Tenth Revision, Clinical Modification
CNV: Choroidal neovascularization
FA: Fluorescein angiography
IRF: Intraretinal fluid
IVI: Intravitreal injection
logMAR: Logarithm of the minimum angle of resolution
nAMD: Neovascular age-related macular degeneration
PED: Pigment epithelial detachment, OCT:optical coherence tomography
SD: Standard deviation
SRF: Subretinal fluid
VA: Visual acuity
